# Sexual Quality of Life in Subjective Tinnitus Individuals With Normal Hearing: A Case–Control Study

**DOI:** 10.1002/brb3.70357

**Published:** 2025-02-19

**Authors:** Beyza Asta, Nazife Öztürk Özdeş, Zehra Aydoğan, Nurcan Uzdil, Rauf Yücel Anadolu, Suna Tokgöz Yılmaz

**Affiliations:** ^1^ Audiology Department, Faculty of Health Sciences Erciyes University Kayseri Turkey; ^2^ Audiology and Speech Disorders Department, Institute of Health Sciences Ankara University Ankara Turkey; ^3^ Audiology Department, Faculty of Health Sciences Ankara University Ankara Turkey; ^4^ Faculty of Health Sciences, Psychiatric Nursing Department Erciyes University Kayseri Turkey; ^5^ Faculty of Medicine, Surgical Medical Sciences, Department of Otorhinolaryngology Ankara University Ankara Turkey

## Abstract

**Introduction:**

This study aimed to assess the quality of sexual life among individuals experiencing subjective tinnitus and to draw comparisons with a control group.

**Methods:**

The study included a group of 21 patients with subjective tinnitus as a case group and 21 healthy individuals as a control group. We used patient information form, Arizona Sexual Experiences Scale (ASEX), Sexual Quality of Life Scale–Female (SQOL‐F), Sexual Quality of Life Scale–Male (SQOL‐M), and Tinnitus Handicap Inventory (THI) as the data collection tool in this research.

**Results:**

The analysis showed no significant difference in average ASEX scores among the groups. It was determined that the SQOL‐F and SQOL‐M score averages were higher in the control group than the case group (*p* < 0.05). A statistically significant inverse correlation was observed between the THI score and the SQOL‐M score among individuals experiencing subjective tinnitus in the case group. A statistically significant effect of tinnitus discomfort obtained by THI was found on sexual quality of life in men. In individuals with tinnitus, 43% of the SQOL‐M scores evaluating the quality of sexual life in men can be explained by THI.

**Conclusion:**

First of all the sexual quality of life scores of case group was worse than healthy individuals. In addition, the negative correlation between THI and sexual quality of life in male indicates that male individuals are more affected. The sexual quality of life in individuals with tinnitus should be specifically addressed by health professionals.

## Graphical abstract

This study highlights that subjective tinnitus negatively affects sexual quality of life. This effect is more pronounced in men, indicating that tinnitus has a more significant impact on their sexual health.

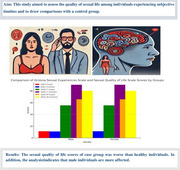

## 1 Introduction

Tinnitus, characterized by the experience of sound in the absence of an external auditory stimulus, is a symptom that is often observed in various populations. The occurrence of tinnitus among adults is relatively high, with approximately 15% of individuals affected (Gilles et al. 2012). Although it does not have a clear pathophysiology, tinnitus is thought to be a result of maladaptive cortical plasticity due to anatomical and functional changes in the neural networks of auditory and non‐auditory systems related to hearing (Shore et al. 2016). In addition, somatosensory, limbic, and autonomic nervous systems are also involved in the development and modulation of tinnitus (Makar 2021).

Studies have shown that 43% of patients with tinnitus have deteriorations in their quality of life (Bauch et al. 2003). Quality of life is often affected in terms of psychological, emotional, and sleep disturbance (Swain 2021). Another important condition that affects the quality of life is sexual health. Sexual health encompasses a holistic state of physical, emotional, mental, and social well‐being in relation to sexuality, and it is recognized as a significant aspect that affects individuals across all age demographics. Many factors such as individuals' age, education level, social structure, general health status, and existing chronic and psychiatric diseases can affect sexuality and sexual health (Taskin Yilmaz et al. 2019).

There are many studies in the literature examining the psychosocial effects of chronic tinnitus on quality of life (Jüris et al. 2013; Onishi et al. 2018; Prabhu and Nagaraj 2020). Studies on tinnitus and sexual health are limited. In a study conducted in Taiwan using a nationwide population‐based database, it was reported that tinnitus was associated with erectile dysfunction (ED) in male individuals (Harrop‐Griffiths et al. 1987). In a study investigating sexual discomfort associated with psychological problems in patients with tinnitus, it was reported that tinnitus affected sexual discomfort in the early stages (Muluk et al. 2007). Another study conducted on male individuals reported that tinnitus generally negatively affected sexual functions (Faul et al. 2009). Some studies in the literature with tinnitus have reported a higher prevalence of sexual problems (Harrop‐Griffiths et al. 1987; Muluk et al. 2007). In this case, it is important to examine the sexual life quality of individuals with tinnitus, and while contributing to the holistic evaluation in the literature, it will also shed light on the treatment and therapy methods that can be created with a multidisciplinary approach. When we take into account this background we aimed to evaluate and compare the sexual quality of life in individuals with and without subjective tinnitus.

The research question addressed in this study is whether subjective tinnitus affects the sexual quality of life in individuals, and how it compares with a control group. A case–control study design was chosen as it allows for a comparison between individuals with subjective tinnitus and healthy controls, providing valuable insights into the impact of tinnitus on sexual health.

## 2 Materials and Methods

### 2.1 Study Design

This research, which is a case–control study, was conducted to determine the effects of tinnitus on the sexual lives of patients with tinnitus and healthy individuals.

### 2.2 Research Sample

To determine the sample size, a pilot study was necessary due to the lack of prior research on sexual quality of life in individuals with subjective tinnitus. Power analysis using GPower 3.1 software (Faul et al. 2009, 2007) was conducted with an effect size of 0.50, resulting in a required sample size of at least 40 participants, with 42 participants ultimately included in the study (21 case group and 21 control group).

### 2.3 Data Collection

In this study, individuals with and without tinnitus who met the inclusion criteria underwent audiologic, and psychoacoustic tinnitus evaluation. The 42 subjects included in this study were divided into two equal groups as case (*n* = 21) and control (*n* = 21) groups according to the presence/absence of subjective tinnitus. Individuals with tinnitus were recruited from those who applied to Ankara University Ibni Sina Hospital Otolaryngology Clinic and were referred to the Audiology, Balance, and Speech Disorders Diagnosis and Rehabilitation Unit. The control group was selected from hospital employees who had similar demographic characteristics to the case group and were volunteers. The case group, consisting of individuals with a partner, had a body mass index (BMI) < 30 kg/m^2^, normal hearing (pure tone thresholds ≤ 20 dB HL and air‐bone gap < 10 dB HL) and immitancemetric findings (Type A tympanogram and acoustic reflexes in the 0.5–4 kHz frequency bands), and complaints of tinnitus lasting more than 6 months (Categories 1–5 on the Tinnitus Handicap Inventory—THI). Obese individuals were excluded from the study because higher BMI is associated with greater impairment in sexual quality of life (Kolotkin et al. 2006).

In contrast to the case group, individuals in the control group had the same inclusion criteria as the case group except for tinnitus. The exclusion criteria were objective tinnitus, systemic disease, regular medication use, middle ear pathologies, acoustic neuroma diagnosis, previous ear or cranial surgery, Meniere's disease, history of sexual or urological surgery, and neuropsychiatric disease. The research data was gathered through in‐person interactions from January 2022 to November 2022. Two participants whose audiologic evaluations could not be completed and five participants who did not complete the questionnaires and scales completely were excluded from the study.

### 2.4 Measurements

In this study patient information form, Arizona Sexual Experiences Scale (ASEX), Sexual Quality of Life Scale–Female (SQOL‐F), Sexual Quality of Life Scale–Male (SQOL‐M), Visual Analog Scale (VAS), and THI were used as data collection tools.

*Patient Information Questionnaire*: This document has been developed by the research team and contains five questions that inquire about the participant's age, gender, educational background, the occurrence of hearing impairment, and any reported issues with tinnitus.

*ASEX*: It is a scale consisting of five items in male and female forms and aimed at determining sexual experience. The scale aims to evaluate sexual functions excluding sexual orientation and relationship with the partner. The total score varies between 5 and 30, with each question being scored from 1 to 6. Low scores suggest a robust, effortless, and fulfilling sexual response, whereas high scores are indicative of greater sexual dysfunction (Soykan 2004). The Cronbach *α* value of the scale is 0.90 (McGahuey et al. 2000). The Cronbach *α* value of the scale was calculated as 0.95 for women and 0.73 for men. In this study, it was calculated as 0.80 for women and 0.68 for men.

*SQOL‐F*: It was developed to determine the level of sexual life quality and validity and reliability studies were conducted. The instrument comprises a total of 18 items, and participants are asked to answer the scale questions by evaluating their sexual lives in the last four weeks. The score range is 18–108, and in order to convert it to 100, the formula (Raw score taken from the scale − 18) × 100/90 is applied. A high total score from the scale means a good quality of sexual life (Symonds et al. 2005). In the adaptation of the scale made in 2010, it was found that the item total score reliability coefficient varied between 0.32 and 0.67 and the Cronbach *α* coefficient for internal consistency was 0.83 (Tugut and Golbasi 2010). In this study, it was found to be 0.97.

*SQOL‐M*: It was created by removing seven items from the Sexual Quality of Life–Female form developed by (Symonds et al. 2005; Abraham et al. 2008). The score obtained from the scale must be converted to 100, for this the formula “(Raw score obtained from the scale − The lowest score that can be obtained from the scale) × 100/(The highest score that can be obtained from the scale − the lowest score)” is used. A high score from the scale indicates that the quality of sexual life is good. The Cronbach *α* was found to be 0.82 (Abraham et al. 2008). The validity and reliability studies of the scale in Turkey were conducted by Kılıç et al. (2018), and the Cronbach *α* was found to be 0.96. In this study, it was found to be 0.90.

*THI*: The Turkish validity and reliability study of the inventory, which was originally developed by Newman et al. (1996), was conducted by Aksoy et al. (2007). The inventory is scored using 4 points for yes, 0 points for no and 2 points for sometimes. The scoring of the inventory is 4 points for a yes answer; 0 points for answering no; Sometimes it is done by using 2 points in the answer. The lowest score that can be obtained in the survey is 0 and the highest score is 100. As the THI score increases, tinnitus‐related distress increases. In addition, there are three subtests in this questionnaire that enable the evaluation of tinnitus in terms of functional, emotional, and catastrophic aspects. The Cronbach *α* coefficient of the inventory was found to be 0.88 (Aksoy et al. 2007). In this study, it was found to be 0.98.

### 2.5 Statistical Analysis

To determine the research sample, studies addressing the sexual quality of life in individuals with normal hearing and subjective tinnitus could not be found. Studies on individuals with subjective tinnitus were examined and a pilot study was needed because they did not meet our hypotheses. Kieser and Wassmer utilized the 80% upper confidence limit method for determining sample size in their research. Their findings indicated that a pilot trial with a sample size ranging from 20 to 40 would effectively reduce the overall sample size required for the main study, which would range from 80 to 250 participants, aligning with standard effect sizes of 0.4 and 0.7. Accordingly, the total sample size of our study was determined as at least 40 individuals and 42 individuals were included in the study, 21 in the case group and 21 in the control group.

Upon completion of the study, a power analysis was conducted to assess the adequacy of the sample size with GPower 3.1. program (Faul et al. 2009). Utilizing an effect size of 0.50 and an alpha error probability of 0.05, the calculated power of the research was found to be 0.88, leading to the conclusion that the sample size was indeed sufficient.

The data were evaluated in the IBM SPSS Statistics Standard Concurrent User V 21 (IBM Corp., Armonk, NY, USA) statistical software. The individuals in both the study and control groups were characterized by their descriptive statistics, including frequency (*n*), percentage (%), and mean ± standard deviation. To assess the normality of the data distribution, the Shapiro–Wilk test was employed, revealing that the data adhered to a normal distribution.

Descriptive variables of the patients in the study and control groups were analyzed using the Pearson chi‐square test in 2 × 2 and rxc tables. The distribution of THI levels of the study group is given as number (*n*) and percentage (%). An independent samples *t*‐test was employed to assess the differences between the groups based on the scales. The Pearson rank correlation coefficient was utilized to evaluate the correlations among the groups in relation to the mean scores of the scales. In addition, Cronbach's *α* coefficients were computed to determine the internal consistency of the scales. The impact of the THI on the SQOL‐M within the study group was analyzed using simple linear regression, with a significance level set at *p* < 0.05 for the comparisons.

## 3 Results

### 3.1 Descriptive Characteristics of Individuals

The individuals in the study were between the ages of 18–55. The average age of individuals for case group with subjective tinnitus was 43.62 ± 7.79; for control group was 40.19 ± 7.68. The groups exhibited comparable characteristics regarding average age, gender distribution, and educational attainment (*p* = 0.159; *p* = 0.753; *p* = 0.980). Table 1 presents a comparative analysis of the groups based on their descriptive characteristics.

**TABLE 1:** Descriptive characteristics of individuals in the case and control group.

	Groups					
	Case (subjective tinnitus) (*n* = 21)		Control (*n* = 21)			
Feature	*n*	(%)	*n*	(%)	Test	*p* value
Age	43.62 ± 7.79	(*x̄* ± SD)	40.19 ± 7.68	(*x̄* ± SD)		*p* = 0.159
Gender						
Male	13	61.9	12	57.1	0.099^a^	0.753
Female	8	38.1	9	42.9		
Education level						
Primary school	2	9.5	2	9.5	0.182^a^	0.980
High school	6	28.6	5	23.8		
Undergraduate	8	38.1	8	38.1		
Postgraduate	5	23.8	6	28.6		
Hearing loss						
Yes					—	—
No	21	100.0	21	100.0		
Tinnitus complaint						
Yes	21	100.0			42.000^a^	**< 0.001**
No			21	100.0		

Abbreviations: SD, standard deviation; *x̄*, mean.

^a^ Chi‐square test was performed.

### 3.2 THI Score and Levels of Individuals

The average THI score of individuals with tinnitus who participated in our study was found to be 41.05 ± 28.88 and the distribution of THI levels is given in Table 2.

**TABLE 2:** Distribution of tinnitus handicap inventory levels of the case group.

		*n*	(%)
THI levels	Very mild (Level 1)	6	28.6
	Mild (Level 2)	4	19.0
	Modarate (Level 3)	4	19.0
	Severe (Level 4)	3	14.3
	Catastropic (Level 5)	4	19.0

Abbreviation: THI, Tinnitus Handicap Inventory.

### 3.3 Comparison of Individuals' ASEX and SQOL Mean Scores According to Groups

Table 3 shows the comparison of individuals' ASEX and SQOL score averages by groups. There is no statistically significant relationship between the ASEX score averages of individuals according to groups (*p* > 0.05). The mean SQOL‐F score of the control group was statistically significantly higher than the case group (*t* = −6.390, *p* < 0.001). In the same way, the mean SQOL‐M score was higher in the control group than in the case group (*t* = −2.458, *p* = 0.023).

**TABLE 3:** Comparison of individuals' Arizona Sexual Experiences Scale and Sexual Quality of Life Scale average scores by groups (*n* = 60).

		Groups		Test statistics	
	Gender	Case (subjective tinnitus)	Control	*t*	*p*
ASEX	Male	11.31 ± 3.75	10.92 ± 1.62	0.343	0.736
	Female	12.75 ± 1.83	11.33 ± 2.83	1.207	0.246
SQOL‐F	Female	55.28 ± 12.51	90.37 ± 9.77	−6.390	**< 0.001**
SQOL‐M	Male	65.59 ± 17.62	80.15 ± 11.59	−2.458	**0.023**

*Note*: Independent samples *t*‐test, mean ± SD.

Abbreviations: ASEX, Arizona Sexual Experiences Scale; SQOL‐F, Sexual Quality of Life Scale–Female; SQOL‐M, Sexual Quality of Life Scale–Male.

### 3.4 Interscale Correlation of Individuals in the Case and Control Groups

Table 4 shows the interscale correlation of individuals in the case and control groups. It was determined that there was a highly (*r* = −0.691; *p* < 0.05) statistically significant and negative relationship between the THI score and the SQOL‐M score of the individuals with subjective tinnitus in the case group but this negative correlation was not significant for women (*r* = −0.530; *p* = 0.177). In the control group, there was no statistically significant relationship between the THI and SQOL scores (*p* > 0.05).

**TABLE 4:** Interscale correlation of individuals in the case and control group (*n* = 42).

	Case		Control
	ASEX	THI	ASEX
ASEX			
*r*		0.391	
*p*		0.080	
SQOL‐M			
*r*	−0.455	−0.691**	−0.325
*p*	0.118	**0.009**	0.303
SQOL‐F			
*r*	−0.301	−0.530	−0.106
*p*	0.468	0.177	0.787

*Note*: Pearson correlation coefficient was used.

Abbreviations: ASEX, Arizona Sexual Experiences Scale; *n*, number of total participants; SQOL‐F, Sexual Quality of Life Scale–Female; SQOL‐M, Sexual Quality of Life Scale–Male; THI, Tinnitus Handicap.

### 3.5 The Effect of the THI on the SQOL‐M by Case Group

The effect of the THI on the SQOL‐M by case group is shown in Table 5. A statistically significant effect of THI was found on SQOL‐M in individuals with tinnitus (*p* = 0.009). When there is a one‐unit increase in THI, there will be a decrease of 0.416 in SQOL‐M. Forty‐three percent of SQOL‐M scores in individuals with tinnitus can be explained by THI.

**TABLE 5:** Effect of Tinnitus Handicap Inventory on the case group Sexual Quality of Life Scale–Male.

		*β* ^1^ (95% CI)	*β* ^2^	*t*	*p*	*F*	*R* ^2^	SE of estimate	Durbin–Watson
Case	Constant	83.211 (68.534/97.887)		12.479	**< 0.001**	10.058	0.430	13.301	2.217
	THI	−0.416 (−0.705/−0.127)	−0.691	−3.171	**0.009**				

## 4 Discussion

The aim of our study is to evaluate the effects of tinnitus on the sexual quality of life and compare it with healthy individuals. For this purpose, surveys regarding both tinnitus and sexual life were applied. In our study, THI was used to assess the impact of tinnitus on individuals' daily lives. ASEX, SQOL‐F, and SQOL‐M questionnaires were used to evaluate the sexual quality of life. While no significant difference was found between the case and control groups in terms of ASEX scores (*p* > 0.05), the mean SQOL‐F and SQOL‐M scores were significantly lower in the case group compared to the control group (*p* < 0.05). This finding suggests that tinnitus negatively affects the sexual quality of life.

The results obtained are similar to previous studies in the literature. Soylu Özler et al. (2014) reported that the International Index of Erectile Function (IIEF) scores were significantly lower in individuals with tinnitus compared to the control group, but there was no significant relationship between the duration and tinnitus discomfort and IIEF scores (Faul et al. 2009). Similarly, Muluk et al. (2007) reported that sexual disturbances may be observed in the early stages of tinnitus, but over time, individuals become accustomed to tinnitus, and do not experience any loss in sexual performance. Our findings differ from these studies in that they show a significant decrease in the quality of sexual life as tinnitus discomfort increases. This may be due to differences in sample size, methods used, or assessment tools.

When examining the effects of tinnitus on sexual life, it is important to evaluate organic and psychosocial factors together. From an organic perspective, the study conducted by Cheng et al. showed that tinnitus may be associated with ED and suggested that tinnitus may be linked to hormonal mechanisms (Cheng et al. 2021; Harrop‐Griffiths et al. 1987). Testosterone, defined as a group of hormones and known as the sexual hormone in men, is known to be related to erectile function, and sexual drive, as well as its role in the protection and development of masculine characteristics (Yafi et al. 2016). It is accepted that tinnitus results from irregular neural synchronization in the auditory pathways. The cochlear nucleus serves as a pivotal center for multisensory integration, receiving auditory input from both the cochlea and the peripheral auditory nerve. This information is subsequently relayed to the auditory cortex through the inferior colliculus and thalamus. Research involving animal models has identified the presence of androgen receptors within the cochlear nucleus of the brainstem, as well as in the cochlear structures of the peripheral auditory system (Chun et al. 2021). These studies indicate that aging and exposure to noise can influence the functionality of androgen receptors in the cochlear nucleus and related cranial nuclei that process other sensory modalities. Such findings imply that a reduction in the activity of these receptors may be linked to diminished mating behaviors observed in animals (Chun et al. 2021). High‐frequency hearing loss has been reported in individuals with hyperandrogenism (Chun et al. 2021). For this reason, it is thought that hormonal changes that trigger frequency‐based changes in the hearing threshold may cause tinnitus. On the other hand, estrogen is thought to have a protective effect on the hearing system. This hormone can regulate blood circulation in the inner ear and show neuroprotective properties. In this direction, it is thought that gender‐specific hormonal changes and tinnitus are two variables that can affect each other. In our study, it was determined that the sexual life quality of both female and male tinnitus individuals was lower than that of normal individuals. In addition, when the neurophysiological model of tinnitus is considered, the possible relationship of hormonal changes with the limbic system may be effective in finding these results. After all, the limbic system is a system that is directly effective in controlling our behaviors in our daily lives. It is possible that a special problem in the limbic system may be related to the sexual life of individuals.

All these findings show that tinnitus is not only an auditory problem, but can also have negative effects on individuals' psychosocial and sexual lives. This situation emphasizes that the quality of sexual life should be taken into consideration in the evaluation and treatment processes of tinnitus. Patients should be supported not only with medical interventions, but also with psychosocial assistance to help them cope with these difficulties. In conclusion, it has been shown that there is a negative relationship between the tinnitus discomfort and the quality of sexual life, and that the quality of sexual life decreases as the tinnitus discomfort increases. These findings reveal that the effects of tinnitus on sexual life should be evaluated and that multidisciplinary approaches are important.

### 4.1 Limitations

This research, which we believe will add value to the existing literature, does have certain limitations. The scope of this single‐center study was restricted by the small sample size. Expanding the reach to broader populations could be achieved through multi‐center studies. Furthermore, the variables analyzed in this investigation are confined to the data collected via the employed measurement instruments. Although factors such as systemic disorders, neuropsychiatric diseases, and hearing disorders are excluded from the study, there may be many conditions that affect the quality of sexual life. Many variables affecting sexuality, such as unrecognized psychological conditions, levels of self‐worth, understanding of sexual matters, inclinations towards specific sexual roles, preferences in sexual partners, and alterations in familial dynamics and general quality of life, have not been tested.

## 5 Conclusion

As a result of this study, it was determined that the mean scores of SQOL‐F and SQOL‐M were higher in the control group than in the case group and these differences were significant. In addition, a statistically significant effect of THI was found on SQOL–M in individuals with tinnitus. A multidisciplinary approach is essential in the evaluation and management of patients with tinnitus. We think that it is necessary to evaluate the patient's tinnitus characteristics and evaluate accompanying symptoms such as subjective hearing loss, otalgia, decreased speech understanding, vertigo, and hyperacusis, as well as taking a systematic detailed anamnesis, including sexual life. We think that if the sexual life quality of individuals with tinnitus is affected, individuals should be evaluated and guided regarding therapy/treatment.

## Author Contributions

**Beyza Asta**: conceptualization, methodology, data curation, resources, writing–review and editing, investigation, project administration, software, validation. **Nazife Öztürk Özdeş**: conceptualization, investigation, writing–original draft, writing–review and editing, visualization, methodology, software, data curation, formal analysis, resources. **Zehra Aydoğan**: methodology, conceptualization, software, supervision, data curation, writing–original draft, project administration. **Nurcan Uzdil**: data curation, conceptualization, methodology. **Rauf Yücel Anadolu**: supervision, writing–review and editing. **Suna Tokgöz Yılmaz**: writing–review and editing, supervision.

## Ethics Statement

Ethical approval of the research was received from Ankara University Faculty of Medicine Human Research Ethics Committee with application number 2022000028 (2022/28), dated January 24 2022 and decision number İ01‐25‐22. In addition, individuals whose consent was obtained through the “Informed Volunteer Consent Form” were included in the study.

## Conflicts of Interest

The authors have no conflicts of interest to declare.

### Peer Review

The peer review history for this article is available at https://publons.com/publon/10.1002/brb3.70357

## Data Availability

Study data can be accessed upon request from the authors.
